# Evaluation of the Relationship Between Nurses' Attitudes Towards Futile Treatments and Their Ethical Attitudes: A Cross‐Sectional and Analytical Study

**DOI:** 10.1111/scs.70035

**Published:** 2025-05-25

**Authors:** Senem Andi, Sumeyye Akcoban, Serap Gungor, Neslihan Yağmur Gider, Betül Tosun, Ezgi Dirgar

**Affiliations:** ^1^ Nursing Department, Faculty of Health Sciences, Hasan Kalyoncu University, Gaziantep, Turkey Mersin Yenişehir District Health Directorate Mersin Turkey; ^2^ Kırıkhan Vocational School, Health Services Department Hatay Mustafa Kemal University Hatay Turkey; ^3^ Vocational School of Health Services Kahramanmaras Sutcu Imam University Kahramanmaras Turkey; ^4^ Ceyhan State Hospital Adana Turkey; ^5^ Hacettepe University Faculty of Nursing Ankara Turkey; ^6^ Faculty of Health Sciences Gaziantep University Gaziantep Turkey

**Keywords:** ethical attitudes, futile treatment, medical futility, nurses, nursing ethics

## Abstract

**Background:**

Futile treatments waste health resources and may conflict with nurses' ethical attitudes, causing moral distress. Nurses frequently administer such treatments due to external pressures.

**Desing and Methods:**

This cross‐sectional study involved 205 nurses in a public hospital in Turkey (June–August 2024). Data were collected using the “Nurse Descriptive Information Form,” “Nurses' Attitudes Towards Futile Treatment Scale (NAFTS),” and “Ethical Attıtude Scale in Nursıng Care (EASNC).”

**Results:**

The mean age of participants was 33.62 ± 7.37 years; 69.8% were female, and 37.1% worked in intensive care. While 90.7% received ethics training, 54.6% lacked knowledge about futile treatments. Over half (54.1%) had administered care they considered futile, with 82.9% citing physician orders as the main reason. Most (70.7%) saw futile treatments as an ethical issue. The NAFTS mean score was 40 ± 7.67 (medium level), and the EASNC score was 158.60 ± 14.85 (high level). A moderate negative correlation (*r* = −0.394, *p* < 0.05) indicated that stronger ethical attitudes reduced acceptance of futile treatments.

**Conclusion:**

As nurses' ethical attitudes strengthened, their tendency to administer futile treatments decreased. Setting realistic, patient‐specific care goals can prevent ethical conflicts and unhelpful care.

**Implications for the Profession:**

Providing ethical training is essential to help nurses avoid futile treatments and make better decisions in challenging situations. Developing supportive policies in healthcare institutions can guide ethical practices and reduce unnecessary treatments. Improving work conditions, such as adjusting hours and reducing workloads, enhances nurses' ability to focus on ethical and patient‐centered care. Encouraging teamwork between nurses and doctors strengthens communication and shared decision‐making, minimising conflicts. Additionally, reducing futile treatments not only saves healthcare resources but also improves the quality of patient care and professional satisfaction for nurses. These measures collectively support the nursing profession and contribute to better healthcare outcomes.

**Reporting Method:**

We adhered to the STROBE.

**No Patient or Public Contribution:**

Given that this study is based on the literature, namely the codes of ethics, there is no direct/indirect involvement of patients or the public.

## 1 Introduction

The development of technology has led to significant advances in healthcare services. In recent years, innovations such as robotic‐assisted surgeries, artificial intelligence‐driven diagnostic tools, and precision medicine have transformed clinical decision‐making and patient management [1, 2]. However, the impact of advancing health technologies on patients' quality of life is controversial. While some studies highlight improvements in early diagnosis and personalised treatment, others argue that aggressive technological interventions may extend biological life without necessarily enhancing well‐being, particularly in critically ill patients [3]. In some cases, it has been observed that only the biological life span is prolonged, but not the quality of life. This situation causes healthcare professionals to face ethical problems, especially in the care and treatment processes given to patients at the end of life [4, 5].

Ethical pressures in healthcare, particularly in end‐of‐life care, contribute to nurses' emotional and professional challenges [6]. Futile treatment refers to care that does not benefit the patient clinically or in terms of quality of life, but continues to be provided [7]. Futile treatment, which is a violation of patients' rights and an important problem for the efficiency of health services, includes not only end‐of‐life care but also all treatment and care processes that do not contribute positively to the patient's quality of life, discharge, or survival [8]. The ongoing use of technologically advanced life‐sustaining treatments in cases where recovery is unlikely has raised concerns about unnecessary suffering, financial burdens, and the ethical justification for prolonging life without meaningful improvement [9]. It is known that the continuation of futile treatment leads to prolonged suffering of patients, restricted access to health services, wastage of health care resources, and burnout among nurses [10]. Therefore, we believe that this process should be carefully considered in order to increase the professional efficiency of nurses and to prevent futile treatment.

Nurses have a key role in regulating futile treatments and ethical decision‐making processes as advocates for patients and their relatives [11]. Preventing futile treatment and improving the organisation of futile treatment in health care institutions can not only improve patient care, but also provide solutions to the ethical issues that nurses face [12]. As healthcare technology advances, nurses are increasingly required to navigate complex ethical dilemmas, including decisions about withholding or withdrawing life‐sustaining interventions based on patient prognosis and quality of life considerations [13]. Nurses' providing care within the framework of ethical principles and values is referred to as ethical attitude. Ethical attitude is an important tool for nurses to solve the problems they face. Adoption and implementation of ethical principles is necessary to alleviate the ethical pressure faced by health professionals in the case of futile treatment [10, 14, 15]. In the literature, there are limited number of studies that examined futile treatment and ethical attitudes together [16, 17]. Mobley et al. [16], in their study on the levels of futile treatment and ethical attitudes in intensive care nurses, found that inadequate ethical attitudes of nurses increased futile treatment attitudes towards patients [16]. In another study, it was reported that providing training to nurses on ethics in certain periods would decrease the level of nurses' futile treatment attitudes [11]. Recent studies emphasise that ethics education combined with technological literacy training can enhance nurses' ability to critically evaluate the use of advanced life‐prolonging treatments in clinical settings [18]. However, it is seen in the results of the research that nurses generally face and experience problems with futile treatment [19, 20, 21, 22]. Kasım and Oflaz [20] found that nurses who believed that futile treatments should be applied under strict rules experienced higher levels of moral distress [20]. Efil et al. [23] reported a positive relationship between intensive care nurses' attitudes towards end‐of‐life care and their ethical attitudes [23]. Furthermore, studies suggest that exposure to advanced life‐support technologies without sufficient ethical training may contribute to increased moral distress among nurses [24]. Yildirim et al. [25] observed that health professionals who frequently witnessed futile treatments opposed such treatments and that death avoidance attitudes increased with the application of futile treatments [25]. Orkun et al. [21] stated that nurses face serious ethical dilemmas in the case of applying futile treatments and have difficulty in solving these dilemmas [21]. These studies emphasise the complex relationship between nurses' attitudes towards futile treatments and their ethical approaches and point to the importance of further research and education in this field.

### 1.1 Aim and Objectives

This study was conducted to examine nurses' attitudes towards futile treatment and their ethical attitudes. In this direction, answers to the following questions were sought:

Is there a significant relationship between the level of ethical attitudes of nurses and the level of attitudes towards futile treatment?

1. Is there a significant relationship between the level of ethical attitudes of nurses and the level of attitudes towards futile treatment?
2. What is the level of nurses' attitudes towards futile treatment?
3. What is the level of nurses' ethical attitude in care?
4. Is there a significant difference between the descriptive characteristics of nurses and the level of attitude towards futile treatment?
5. Is there a significant difference between the descriptive characteristics of nurses and the level of ethical attitude in care?

## 2 Methods

### 2.1 Desing

A cross‐sectional analytical study.

### 2.2 Sample

The study was conducted between June and August 2024 in a public hospital in the Mediterranean region of Turkey, and the population consisted of 958 nurses. The sample size was calculated using the G*Power 3.1.9.2 program with a 95% confidence interval and 80% power, assuming a moderate correlation between the two scales (*r* = 0.3, [26]), and was determined to be at least 174 people. Considering the possibility of data loss (15%) and the possibility of incomplete completion of online questionnaires, 400 nurses who actively participated in patient care were reached; 167 responded incompletely/never, and 28 refused to participate. The study was finalised with the participation of 205 nurses. The study employed a convenience sampling method, where participants were selected based on their accessibility and willingness to participate. Inclusion criteria included being a registered nurse actively working as a nurse for at least 6 months and in direct patient care during the study period, while nurses who were on leave or in administrative roles were excluded. Efforts were made to encourage participation, including reminder emails, flexible survey completion windows, and anonymisation assurances to increase response rates. STROBE checklist was followed for reporting in the study.

### 2.3 Data Collection

The study data were collected through “Google Forms”, a reliable online platform. We also anticipated that relying solely on Google Forms could limit the participation of nurses who are comfortable with digital tools and potentially exclude those who are less familiar with the technology. To mitigate this issue, we provided detailed instructions and technical support to ensure accessibility at each stage of data collection. Before data collection, a pilot study was conducted with 20 nurses before the main data collection phase to assess the comprehensibility and clarity of the survey instruments. The results of the pilot study were not included in the final analysis, as its primary purpose was to refine the methodology. However, insights gained from the pilot study helped in improving the wording of certain survey questions and ensuring the reliability of the data collection process. These refinements contributed to enhancing the validity of the final study design. In addition, in order to ensure the reliability of the data, inconsistent responses were identified by adding similar questions to certain sections of the questionnaire as a control. The questionnaire took approximately 10 min to complete. Three measurement instruments were used to collect data in the study.

### 2.4 Instruments

#### 2.4.1 Nurse Descriptive Information Form

The questionnaire was prepared by the researchers by reviewing the literature and included 15 questions about nurses' individual characteristics, futile treatment, and ethical attitudes [5, 14, 17, 27].

#### 2.4.2 Nurses' Attitudes Towards Futile Treatment Scale (NAFTS)

The scale was developed by Yıldırım et al. in 2019 and consists of 18 items and 4 sub‐dimensions. The sub‐dimensions of the scale are identification (beliefs), decision‐making, ethical principles and law, dilemmas and responsibilities, and the scale is a five‐point Likert scale (1 = Strongly agree, 2 = Agree, 3 = Neither agree nor disagree (undecided), 4 = Disagree, 5 = Strongly disagree). Scores between 18 and 90 can be obtained from the scale, and lower scores indicate that unhelpful interventions should not be performed, while higher scores indicate an attitude that unhelpful interventions can be performed appropriately. Items 12 and 15 in the scale are reverse scored, which could potentially lead to response confusion. To mitigate this, clear instructions were provided to participants. Additionally, a pilot study was conducted to ensure the clarity of reverse‐scored items, and inconsistent responses were checked to improve reliability. Cronbach's alpha value of the scale was found to be 0.72 [5], while in this study, it was found to be 0.76, indicating an acceptable reliability level.

#### 2.4.3 Ethical Attıtude Scale in Nursıng Care (EASNC)

In the scale developed by Özçiftçi and Akın in 2020, a five‐point Likert scale (1: Strongly Disagree, 2: Disagree, 3: Undecided, 4: Agree, 5: Strongly Agree) has 34 items. The scale, developed by Özçiftçi and Akın in 2020, consists of 34 items and is structured as a five‐point Likert scale (1: Strongly Disagree, 2: Disagree, 3: Undecided, 4: Agree, 5: Strongly Agree). The scale is scored between 34 and 170, with higher scores indicating a more positive ethical stance. Cronbach's alpha value of the scale was found to be 0.96 [14], and in this study, it was found to be 0.99, demonstrating a very high level of internal consistency and reliability.

### 2.5 Data Analysis

Statistical analysis was conducted using IBM SPSS 25 software (IBM Corporation, Armonk, NY). Descriptive statistics, including means, standard deviations, frequencies, and percentages, were used to summarise the data. The Shapiro–Wilk test was performed to assess normality. For comparisons between groups, the Mann–Whitney *U* test and Kruskal–Wallis test were used for non‐parametric variables; Spearman correlation analysis was used to examine relationships between continuous variables. Multiple comparisons were corrected using the Bonferroni correction method. A significance level of *p* < 0.05 was considered statistically significant for all statistical decisions.

### 2.6 Ethical Considerations

Ethical approval (date: 21/05/2024, decision no: 27) was obtained from the ethics committee of a university to which one of the authors was affiliated before starting the study. Before starting the online survey, the nurses were informed about the research and their online consent was obtained. The research was conducted in accordance with the principles of the Declaration of Helsinki and the data of the participants were kept confidential. The authors declare that they have no competing interests.

## 3 Results

The mean age of the nurses included in the study was 33.62 ± 7.37 years and 69.8% were female. Of the participants, 86.8% had a bachelor's degree, 24.9% had been working as a nurse for 1–4 years, and 39.5% had been working in their current institution for 3–5 years. While 72.2% of the nurses worked day/night shifts, 42.4% worked between 48 and 60 h per week. The rate of receiving training on ethics after graduation was found to be 90.7%, whereas 54.6% stated that they did not have information about futile treatment. 54.1% of the participants stated that they had previously applied a treatment or care that they thought was useless. While 78.5% of those who applied futile treatment stated that the harm of these applications was more than the benefit, 20% stated that they applied futile treatment once a week. 82.9% of the nurses stated “physician's order” as the reason for continuing these practices, and 70.7% considered futile treatment as an ethical problem. In addition, 11.2% considered useless treatments and practices as an ethical problem in terms of economic loss. The mean NAFTS score of the nurses was 40 ± 7.67 and the mean EASNC score was 158.60 ± 14.85. According to the Shapiro–Wilk normality test, the scales are not normally distributed (*p* < 0.05) (Table 1).

**TABLE 1:** Descriptive characteristics of nurses (*N* = 205).

Characteristic		
Age, mean ± SD	33.62 ± 7.37	
Gender, *n* (%)		
Female	143	69.8
Male	62	30.2
Education, *n* (%)		
Undergraduate	13	6.4
Bachelor	178	86.8
Postgraduate	14	6.8
Working time, *n* (%)		
1–4 year	51	24.9
5–9 year	47	22.9
10–18 year	57	27.8
> 19 year	50	24.4
Employment duration, *n* (%)		
1–2 year	66	32.2
3–5 year	81	39.5
> 6 year	58	28.3
Department, *n* (%)		
Intensive care unit (ICU)	76	37.1
Emergency service (ES)	42	20.5
Internal medicine ward (IMW)	33	16.1
Surgical ward (SW)	32	15.6
Polyclinic	22	10.7
Work schedule, *n* (%)		
Daytime only	57	27.8
Day/night	148	72.2
Weekly average working hours, *n* (%)		
40–42 h	82	40.0
44–46 h	36	17.6
48–60 h	87	42.4
Have you ever had training on ethics after graduation?, *n* (%)		
Yes	186	90.7
No	19	9.3
Do you know about futile treatment?, *n* (%)		
Yes	93	45.4
No	112	54.6
Have you ever given care or treatment you thought was futile?, *n* (%)		
Yes	111	54.1
No	94	45.9
Examples of futile treatments applied^a^, *n* (%)		
Treatments where the harm outweighs the benefit	161	78.5
All treatments given to patients with brain death	28	13.7
Treatments provided to terminal‐stage cancer patients	10	4.8
How often do you provide futile treatments?^a^, *n* (%)		
Every day	20	9.8
Once a week	41	20.0
Once a month	24	11.6
Rarely	36	17.6
What are the reasons for continuing futile treatments and practices?^b^, *n* (%)		
Attitude of hospital management	23	11.2
Legal obligations	95	46.1
Patient's family	61	29.8
Physician's order	170	82.9
Beliefs and values of the physician/nurse	40	19.5
Do you think providing futile treatment and care is an ethical issue?, *n* (%)		
Yes	145	70.7
No	60	29.3
Why do you think providing futile treatment and care is an ethical issue?^a^, *n* (%)		
Lack of expected response to treatments	21	10.2
Economic loss	23	11.2
Prolongation of the patient's suffering	8	3.9

Scales	Cronbach's alpha	Ort ± SD (min‐max)	95% CI for difference	
			Lower	Upper
NAFTS	0.769	40.00 ± 7.67 (18–90)	39.00	41.11
EASNC	0.990	158.60 ± 14.85 (34–170)	156.55	160.64

Test of normality according to Shapiro–Wilk			
	Statistic	df	Sig.
NAFTS total	0.975	205	0.001
EASNC total	0.726	205	0.000

^a^ Not all participants responded.

^b^ Multiple answers were given.

A moderate and negative significant relationship was found between NAFTS and EASNC (*r* = −0.394, *p* < 0.05). As NAFTS score increases, EASNC score decreases. Among the sub‐dimensions of NAFTS, there was a moderate negative correlation between “identification” and EASNC (*r* = −0.420), a weak negative correlation with “decision making” (*r* = −0.215) and a weak negative correlation with “ethical principles and law” (*r* = −0.321) (*p* < 0.05) (Table 2).

**TABLE 2:** The Relationship Between NAFTS and EASNC Scores of Nurses (*N* = 205).

		EASNC	NAFTS identification	NAFTS decision making	NAFTS ethical principles and law	NAFTS ethical dilemma and responsibility	NAFTS total score
EASNC	*r* *p*	1					
NAFTS Identification	*r* *p*	−0.474 < 0.001	1				
NAFTS Decision Making	*r* *p*	−0.252 < 0.001	0.377 < 0.001	1			
NAFTS Ethical Principles and Law	*r* *p*	−0.403 < 0.001	0.632 < 0.001	0.306 < 0.001	1		
NAFTS Ethical Dilemma and Responsibility	*r* *p*	−0.040 0.567	0.187 0.007	0.209 < 0.001	0.093 0.183	1	
NAFTS Total Score	*r* *p*	−0.455 < 0.001	0.918 < 0.001	0.594 < 0.001	0.661 < 0.001	0.446 < 0.001	1

*Note: r*, Spearman Correlation Coefficient, *p* < 0.001. A moderate and negative significant relationship was found between NAFTS and EASNC (*r* = −0.455, *p* < 0.05). As NAFTS score increases, EASNC score decreases. Among the sub‐dimensions of NAFTS, there was a moderate negative correlation between “identification” and EASNC (*r* = −0.474), a weak negative correlation with “decision making” (*r* = −0.252) and a moderate negative correlation with “ethical principles and law” (*r* = 0.403) (*p* < 0.05) (Table 2).

There were no statistically significant differences between the descriptive characteristics of the participants, such as age, gender, and educational status, and the total scale scores, whereas statistically significant differences were found between some other variables, such as the duration of employment, the unit of employment, and the type of work. In the advanced statistics with Bonferroni correction, it was found that the scores of nurses working 5–9 years were higher than those working 1–4 years and 10–18 years (*x* ^2^ = 10.389, *p* = 0.016). NAFTS scores of nurses working in polyclinics were found to be significantly lower than those working in intensive care (*x* ^2^ = 15.217, *p* = 0.004). NAFTS scores of nurses working only during the day were found to be lower than those working in day/night shifts (*z* = −2.420, *p* = 0.016). The scores of nurses working 48–60 h per week were significantly higher than those working less (*x* ^2^ = 16.298, *p* < 0.001). The NAFTS scores of nurses who stated that they did not receive ethical training were significantly higher than those who received ethical training (*z* = −3.158, *p* = 0.002). In addition, the scores of those who did not think that they practiced futile treatment were lower than those who thought that they practiced such practices (*z* = −6.992, *p* < 0.001). EASNC scores of nurses working in the internal ward were significantly higher than those working in the polyclinic (*x* ^2^ = 14.331, *p* = 0.006). It was found that the scores of those working 44–46 h per week were higher than those working 48–60 h per week (*x* ^2^ = 11.912, *p* = 0.003). The scores of those who received ethical training were significantly higher than those who did not (*z* = −4.025, *p* < 0.001). The scores of those who practiced futile treatment were higher than those who did not (*z* = −4.448, *p* < 0.001) (Table 3).

**TABLE 3:** Comparison of scale scores according to descriptive characteristics (*N* = 205).

Characteristic	NAFTS		Test (z. *x* ^2^) *p*	EASNC		Test (z. *x* ^2^) *p*
	Mean ± SD	Median (IQR)		Mean ± SD	Median (IQR)	
Age						
< 27 years	39.7391 ± 7.57	40.50 (13.00)	*x* ^2^ = 2.451 *p* = 0.484	155.91 ± 15.70	165.0 (32.2)	*x* ^2^ = 6.758 *p* = 0.080
28–32 years	39.08 ± 7.04	40.5 (9.00)		161.50 ± 14.24	170.0 (14.0)	
33–39 years	41.14 ± 8.07	43.0 (11.0)		159.53 ± 14.18	168.0 (22.0)	
> 40 years	4047 ± 8.10	40.0 (11.50)		156.88 ± 15.08	167.0 (32.5)	
Gender						
Female	40.65 ± 7.84	41.0 (12.0)	z = −1.840 *p* = 0.066	158.58 ± 14.92	168.0 (29.0)	z = −0.606 *p* = 0.545
Male	38.69 ± 7.13	38.50 (9.5)		158.66 ± 14.78	169.0 (30.25)	
Education						
Undergraduate	44.23 ± 10.38	41.0 (21.0)	*x* ^2^ = 5.449 *p* = 0.066	150.53 ± 17.27	136.0 (33.5)	*x* ^2^ = 4.835 *p* = 0.089
Bachelor	39.47 ± 7.20	40.0 (11.0)		158.91 ± 14.90	169.0 (30.0)	
Postgraduate	43.64 ± 8.99	45.0 (11.5)		162.21 ± 8.86	166.0 (13.25)	
Working time						
1–4 years^a^	39.31 ± 7.55	40.0 (13.0)	*x* ^2^ = 1.280 *p* = 0.734	155.60 ± 16.10	167.0 (33.0)	*x* ^2^ = 10.389 *p =* 0.016* (a−b) (c−b)
5–9 years^b^	39.61 ± 6.85	40.0 (9.0)		164.32 ± 10.73	170.0 (5.0)	
10–18 years^c^	40.91 ± 7.89	42.0 (11.5)		158.01 ± 15.32	167.0 (30.0)	
> 19 years^d^	40.28 ± 8.35	40.0 (13.25)		157.06 ± 15.31	168.0 (15.3)	
Employment duration						
1–2 years	39.50 ± 7.12	40.0 (10.2)	*x* ^2^ = 0.836 *p* = 0.658	155.65 ± 15.72	166.50 (32.5)	*x* ^2^ = 1.827 *p* = 0.401
3–5 years	39.95 ± 7.68	41.0 (11.5)		159.91 ± 14.09	169.0 (25.0)	
> 6 years	40.86 ± 8.29	40.5 (13.2)		160.13 ± 14.60	169.0 (13.25)	
Department						
ICU^a^	38.14 ± 6.76	38.0 (9.75)	*x* ^2^ = 15.217 *p* = 0.004* (a−e)	159.82 ± 14.31	169.0 (26.5)	*x* ^2^ = 14.331 *p* = 0.006* (e−c)
ES^b^	39.97 ± 8.11	42.0 (13.25)		155.64 ± 15.64	166.5 (33.2)	
IMW^c^	39.36 ± 7.13	40.0 (10.30)		164.39 ± 12.53	170.0 (1.50)	
SW^d^	42.31 ± 8.36	42.5 (12.75)		156.71 ± 16.41	167.5 (32.2)	
Polyclinic^e^	44.63 ± 7.41	45.0 (11.75)		154.09 ± 13.90	157.0 (30.7)	
Work schedule						
Daytime only	42.24 ± 8.40	43.0 (12.5)	*z* = −2.420 *p* = 0.016*	158.43 ± 14.65	169.0 (30.5)	*z* = −0.090 *p* = 0.929
Day/night	39.22 ± 7.22	40.0 (10.7)		158.66 ± 15.97	169.0 (30.0)	
Weekly average working hours						
40–42 h^a^	41.65 ± 8.02	43.0 (12.2)	*x* ^2^ = 16.298 *p* < 0.001** (b−c) (c−a)	158.47 ± 15.72	170.0 (32.5)	*x* ^2^ = 11.912 *p* = 0.003 (c−b)
44–46 h^b^	35.91 ± 6.07	34.0 (8.0)		164.19 ± 14.09	170.0 (25.0)	
48–60 h^c^	40.27 ± 7.36	40.0 (12.0)		156.41 ± 14.60	164.0 (13.25)	
Have you ever had training on ethics?						
Yes	39.53 ± 7.66	40.0 (10.5)	*z* = −3.158 *p* = 0.002*	159.68 ± 14.52	169.0 (28.2)	*z* = −4.025 *p* < 0.001**
No	45.11 ± 5.67	46.0 (7.0)		148.05 ± 14.21	147.0 (25.0)	
Do you know about futile treatment?						
Yes	39.56 ± 8.04	40.0 (12.0)	*z* = −1.095 *p* = 0.274	158.50 ± 14.75	169.0 (28.5)	*z* = −0.051 *p* = 0.959
No	40.47 ± 7.36	41.0 (12.0)		158.65 ± 14.99	169.0 (31.7)	
Have you ever given care or treatment you thought was futile?						
Yes	36.52 ± 6.09	35.0 (10.0)	*z* = −6.992 *p* < 0.001**	162.41 ± 12.82	170.0 (8.0)	*z* = −4.448 *p* < 0.001**
No	44.24 ± 7.24	44.0 (9.2)		154.10 ± 15.86	159.5 (34.0)	
Examples of Futile Treatments Applied*						
Treatments where the harm outweighs the benefit	39.07 ± 6.54	40.5 (9.5)	*x* ^2^ = 3.641 *p* = 0.162	159.17 ± 14.10	166.50 (28.7)	*x* ^2^ = 1.183 *p* = 0.554
All treatments given to patients with brain death	41.33 ± 4.41	41.5 (8.7)		156.66 ± 15.02	163.5 (28.7)	
Treatments provided to terminal‐stage cancer patients	35.00 ± 7.21	34.5 (15.5)		161.10 ± 11.92	166.5 (11.9)	
How often do you provide futile treatments?*						
Every day^a^	36.40 ± 6.42	35.0 (8.7)	*x* ^2^ = 23.209 *p* < 0.001** (b−d) (c−d) (a−d)	160.20 ± 14.92	170.0 (29.5)	*x* ^2^ = 20.167 *p <* 0.001** (d−b) (d−c)
Once a week^b^	34.46 ± 5.08	34.0 (6.0)		165.80 ± 10.06	170.0 (1.0)	
Once a month^c^	35.87 ± 6.29	35.0 (12.0)		164.04 ± 11.75	170.0 (5.5)	
Rarely^d^	41.88 ± 6.59	44.0 (9.25)		156.77 ± 14.46	164.0 (29.7)	
Do you think providing futile treatment and care is an ethical issue?						
Yes	37.97 ± 6.95	37.0 (10.0)	z = −5.956 *p* < 0.001**	161.23 ± 13.82	170.0 (12.5)	z = −4.061 *p* < 0.001**
No	45.10 ± 6.98	45.0 (10.3)		152.25 ± 15.42	151.5 (33.7)	
Why do you think providing futile treatment and care is an ethical issue?*						
Lack of expected response to treatments^a^	42.28 ± 7.51	41.0 (9.0)	*x* ^2^ = 7.955 *p* = 0.019* (b−a)	153.52 ± 12.95	158.0 (27.5)	*x* ^2^ = 15.478 *p* < 0.001** (a−b)
Economic loss^b^	36.35 ± 7.74	35.0 (11.0)		163.73 ± 12.72	170.0 (3.0)	
Prolongation of the patient's suffering^c^	36.12 ± 5.35	34.5 (9.25)		163.75 ± 11.71	169.5 (8.2)	

*Note: z*: Mann Whitney *U* test *x* ^2^: Kruskall–Wallis test. a, b, c, d, e Post Hoc Bonferroni.

* *p* < 0.05.

** *p* < 0.001.

## 4 Discussion

This study, which was conducted with 205 nurses to evaluate the relationship between nurses' attitudes towards futile treatment for care and their ethical attitudes, found that there was a negative relationship between nurses' ethical attitudes in care and their attitudes towards futile treatment; it was determined that as ethical attitudes increased, attitudes towards futile treatment decreased. Nurses should be aware of the importance of the harm and ethical problems that may be caused to patients in futile treatment practices [10]. Since ethical principles such as autonomy and non‐harm are in question as a result of futile care practice, the increase in nurses' ethical attitudes may lead to a decrease in futile treatment attitudes. In particular, negative relationships were found between the “identification” sub‐dimension of NAFTS and ethical attitudes at a moderate level with “decision making” at a weak level and with “ethical principles and law” at a weak level.

It was determined that nurses' ethical attitudes decreased in decision‐making about futile treatment. Nurses should include the values of the patient in the decision‐making process regarding the initiation or termination of futile treatments [28]. It can be said that the fact that nurses, who are the patient's advocates, do not take part in the decision‐making process of the patient and his/her family and have to implement decisions that they do not agree with leads to ethical problems. It was found that the ethical attitude of nurses who defined futile treatment and evaluated it in terms of ethical principles and law decreased. Ethical and legal principles should be taken into consideration regarding futile treatments [5]. The legal dimension of useless care given to the patient may increase the stress on employees. Therefore, when nurses evaluate useless treatment within the scope of ethical principles and law, it can be thought that their ethical attitude decreases due to the stress on them. These findings suggest that improving nurses' ethical attitudes may reduce their attitudes towards futile treatment and enable them to make more informed decisions. Futile treatment practices create serious ethical dilemmas for nurses and this situation directly affects the quality of patient care [21]. Nurses have a critical role in patient advocacy and ensuring that patients receive the best care. However, this role requires not only clinical skills but also ethical awareness and decision‐making skills [10]. It has been supported by previous research that attitudes towards futile treatment lead to both professional burnout and decreases in the quality of patient care [11, 16]. These results indicate that improvements should be made at individual and institutional levels to increase the ethical awareness of nurses and to prevent futile treatment practices. In this context, it is critical for healthcare institutions to adopt policies that support ethical decision‐making processes and to establish training programs to improve the professional competencies of nurses. Strengthening ethical attitudes will both increase the professional satisfaction of nurses and positively affect the quality of patient care. In this study, it was found that the ethical attitudes of nurses who considered futile treatment as an ethical problem and an economic loss were higher. Borhani et al. [19] also emphasised that futile care triggers conditions that may lead to ethical problems in nurses [19]. Similarly, another study reported that futile treatments cause serious ethical problems for nurses, which has a negative impact on the overall performance of nurses, patients and healthcare organisations [11]. These effects are not only limited to burnout and professional dissatisfaction at the individual level, but are also understood to cause waste of resources in health systems and violation of patient rights. In this case, preventing futile treatment practices and preventing the emergence of ethical problems necessitates improvements at individual and systemic levels. It is thought that futile treatments increase ethical dilemmas and the solution of these dilemmas can be possible by establishing training and support mechanisms that will improve the professional competence of nurses. Moreover, potential confounding factors such as nurses' demographic characteristics, work environment, and institutional policies should be considered when interpreting these findings. For example, age, education level, and years of experience may influence ethical attitudes and perceptions of futile treatments [29]. Additionally, cultural and organisational factors, including hospital management practices and ethical guidelines, might impact nurses' decision‐making processes [9]. Addressing these variables is crucial to providing a comprehensive understanding of the study results.

In the present study, it was concluded that nurses working in polyclinics had higher futile treatment attitudes than nurses working in intensive care. In another study, it was reported that nurses working in neonatal intensive care were sensitive about futile treatments [11]. In polyclinic services, nurses mostly serve outpatients and patients whose condition is not critical. The fact that nurses working in intensive care have lower attitudes towards futile care may be thought to be due to the fact that they provide direct care to patients in the critical period of their lives and closely follow their suffering process. Nurses are prone to adopt a care approach that centres on the patient's quality of life. This may lead them to be more critical of futile treatments. The study also suggests that working conditions, such as shift patterns and weekly working hours, may act as confounding variables affecting both ethical attitudes and clinical practices [24]. Nurses working longer hours (48–60 h per week) showed a higher tendency towards futile treatments, which may be attributed to increased fatigue and reduced decision‐making capacity [22]. Future studies should control for these factors to ensure more accurate interpretations of the relationship between ethical attitudes and futile treatment practices.

We found that nurses working only in day shifts had higher attitudes towards futile treatment compared to nurses working in shift shifts. This provides important clues about how nurses' working patterns and hours may affect their ethical and caring attitudes. Although nurses working in day shifts generally have a more regular work schedule, it can be thought that their workload and responsibilities are more intense and therefore they may become more sensitive to futile treatment practices. Begjani et al. [11] found that nurses working in shifts had higher levels of futile care attitudes than those working only in day shifts [11]. Factors such as fatigue and insomnia experienced during shift work may affect the decision‐making processes of nurses and cause them to be more careless about futile treatments for self‐protection. This may lead to the adoption of futile treatment practices by reducing the tendency to question the treatment given. On the other hand, when the relationship between weekly working hours and futile treatment attitudes was examined, it was observed that nurses working 48–60 h per week had higher futile treatment attitudes compared to those working 44–46 h per week. This finding suggests that long working hours may negatively affect nurses' physical and mental health and weaken their attitudes towards ethical decision‐making and patient care. The findings of Voultsos et al. [22] clearly show the impact of working hours and working arrangements on nurses' ethical decision‐making and patient care attitudes [22]. It is thought that nurses' attitudes towards futile care and treatment may change positively with the appropriate regulation of working hours and shift arrangements. Positive attitudes of nurses towards ethical problems have an important effect on solving ethical dilemmas that may be encountered in patient care and increasing the quality of health services [30]. In this research, it was found that nurses' ethical attitudes towards care were at a high level. Similarly, it is reported in the literature that nurses' ethical attitudes are generally high and their awareness of ethical principles and patient rights is strong [29, 31]. This shows nurses' commitment to ethical principles and their understanding of patient‐centered care while fulfilling their professional responsibilities. In this study, the finding that the ethical attitudes of nurses who stated that they received ethical training were higher supports this view.

It was observed in this study that nurses who worked 5–9 years in the profession had higher ethical attitudes than nurses with less work experience and nurses with more years of work experience. Kirca and Özgönül [32] reported in their study that nurses with shorter working years had higher ethical attitudes [32]. In another study, it was concluded that nurses who worked 1–10 years in the profession had higher ethical sensitivity than nurses with more years of professional service [33]. In the current study, nurses with 5–9 years of experience in the profession may represent a group that has generally gained sufficient knowledge and skills for the nursing profession, but has not yet encountered negative effects such as professional burnout or monotony. During this period, nurses may tend to maintain their professional motivation and adhere to ethical principles. While less experienced nurses may still be in the development stage in terms of professional knowledge and ethical awareness, factors such as workload, burnout, and routinisation may negatively affect ethical attitudes in nurses who have been working for a longer period of time.

In addition, in this study, it was found that the ethical attitudes of nurses who received training on ethical issues after graduation were high. This finding suggests that ethical training plays an important role in increasing nurses' professional ethical awareness and enabling them to make stronger ethical decisions in patient care. Similarly, it has been reported in the literature that ethical training positively affects ethical attitudes in nursing care [34]. It has been stated that ethical training improves nurses' ability to recognise the ethical problems they face and to produce solutions, and also enables them to access information sources more easily when making decisions on ethical problems [29]. Increasing nurses' access to ethical training, improving their professional ethical awareness and enabling them to integrate this knowledge into practice are of great importance in terms of increasing the quality of patient care and professional satisfaction.

## 5 Limitations

The fact that the study was conducted in a single public hospital may limit the generalizability of the findings to nurses working in private healthcare institutions or different regional contexts. Although a sample size calculation was performed during the planning phase of the study, the final participation rate represented 21.3% of the target population. This rate was influenced by factors such as workload, shift schedules, and individual availability, which may have introduced selection bias. Future studies should consider strategies such as incentives, more flexible data collection methods, and targeted recruitment approaches to increase participation rates.

Additionally, the reliance on self‐reported data poses the risk of response bias, as participants may have provided socially desirable responses rather than fully accurate reflections of their attitudes and behaviours. Future research should incorporate objective data collection methods, such as direct observations or structured interviews, to enhance the validity of findings.

Although nurses quickly adapt to modern technology and actively use it in the workplace, the use of Google Forms requires a certain level of digital literacy, which may have introduced selection bias. While efforts were made to support participants throughout the process, future studies should explore alternative data collection strategies to ensure broader accessibility and inclusivity.

## 6 Conclusions

It was found that nurses' tendency to apply futile treatment decreased with increasing ethical attitudes, and ethical education increased awareness of futile treatment and improved their skills to cope with ethical problems. On the other hand, nurses who did not receive ethical training were found to have higher attitudes towards futile treatment. The study shows that long working hours and inadequate support negatively affect both professional efficiency and quality of patient care. It was determined that intensive care nurses were more sensitive to futile treatment with the experience they gained in critical patient care. In conclusion, the importance of continuing education programmes to increase the ethical awareness of nurses is emphasised. Improving working conditions and developing policies to support ethical decision‐making processes will be effective in improving the quality of patient care.

### 6.1 Relevance to Clinical Practice

This study highlights the critical role of ethical attitudes in reducing futile treatment practices among nurses, providing actionable insights for clinical practice. By fostering ethical awareness and decision‐making skills through targeted training programmes, healthcare institutions can improve the quality of patient care while minimising moral distress and resource wastage. The findings underscore the importance of tailoring policies to support ethical decision‐making and optimising work conditions, such as regulating shift patterns and reducing excessive workloads, to enhance professional efficiency and care standards. Strengthening ethical principles in nursing practice not only boosts job satisfaction but also ensures patient‐centred care, making it a priority for sustainable healthcare systems.

## Author Contributions

Study design: Senem Andi, Betül Tosun. Data collection: Senem Andi, Neslihan Yağmur Gider. Data analysis: Senem Andi, Betül Tosun. Study supervision: Senem Andi, Betül Tosun, Sumeyye Akcoban, Serap Gungor, Ezgi Dirgar. Manuscript writing: Senem Andi, Betül Tosun, Sumeyye Akcoban, Serap Gungor, Ezgi Dirgar, Neslihan Yağmur Gider.

## Ethics Statement

Prior to initiating the study procedures, approval was obtained from the non‐interventional research ethics committee of Hatay Mustafa Kemal University (Date: 21.05.2024, Number of Decisions: 27, Number of Meetings: 05). Permission was also granted by the health institution where the research was conducted. Patient confidentiality was prioritised within the scope of the study. Oral and written consent was obtained from patients, and they were assured that the information collected would be used solely for research purposes. The principles of the Helsinki Declaration were followed during the entire process. Permission to use the scales, whose validity and reliability were previously established by their authors, was obtained via email.

## Conflicts of Interest

The authors declare no conflicts of interest.

## Data Availability

The data that support the findings of this study are available from the corresponding author upon reasonable request.
